# Supplementary material for: A statistical method for removing unbalanced trials with multiple covariates in meta-analysis
Source: PLoS One. 2023 Dec 15;18(12):e0295332. doi: 10.1371/journal.pone.0295332 (PMC10723740; doi:10.1371/journal.pone.0295332)
Supplement: S1 Appendix — (PDF) [file pone.0295332.s001.pdf]

S1 Appendix

Aims

The aim of the simulation is to evaluate the suitability of the proposed procedure to identify the unbalanced trials in terms of covariate imbalance in different scenarios. The simulation does not take into account any potential effect that the covariate (mean age) may have on the response variable.

Scenarios

The simulation comprises 1000 repetitions, conducted across 9 distinct scenarios involving 1 covariate, the *mean age*. These scenarios aim to replicate the *Hep* dataset as presented in the paper. Each scenario consists of:

- 25 trials with 2 arms, the control (*ctrl*) and experimental (*exp*) arms,
- each trial includes 200 patients, equally divided between the *exp* and *ctrl* arms (i.e., 100 patients per arm),
- the scenarios are differentiated by varying levels of imbalance, determined by:
  - 3 pairs of means for the 2 arms [(50, 50); (55, 50); (60, 50)]. These pairs of means mimic two *Normal* populations with "equal" "quite different", and "very different" means.
  - each pair of means is generated with 3 different standard deviation values: 5, 10, and 15, to simulate different *age* distributions.

Data is generated for each scenario using a two-step procedure:

1. generate 25 *age* means in each arm, $\mu_{i(ctrl)}$ and $\mu_{i(exp)}$ (i = 1, 2, …, 25), from normal distributions with parameters detailed in Table B1:

Table 1. Simulation's scenarios with 3 mean values (50; 55; 60) and 3 standard deviations (5; 10; 15) for the *mean age* in the two arms.

	Arm	
Scenario	*Ctrl* – $N(\mu, \sigma)$	*Exp* – $N(\mu, \sigma)$

1	μ~N(50, 5)	μ~N(50, 5)
2	μ~N(50, 10)	μ~N(50, 10)
3	μ~N(50, 15)	μ~N(50, 15)
4	μ~N(55, 5)	μ~N(50, 5)
5	μ~N(55, 10)	μ~N(50, 10)
6	μ~N(55, 15)	μ~N(50, 15)
7	μ~N(60, 5)	μ~N(50, 5)
8	μ~N(60, 10)	μ~N(50, 10)
9	μ~N(60, 15)	μ~N(50, 15)

2. In each of the 25 trials, 100 age values are generated for both control and experimental groups [$x_{ij(ctrl)}$ and $x_{ij(exp)}$; i = 1, 2, …, 25; j = 1, 2, …, 100)]. These values are drawn from normal distributions with means derived from the previous simulation. In this case, the standard deviations used in this step are set at double the magnitude of those previously specified, because the units present higher variability than their means.

Targets

The greater the difference between the means, the greater the number of trials removed and the larger the standard deviation, the greater the number of trials removed.

Methods

The proposed procedure to assess the covariate balance between the meta-arms used in this simulation is the same one presented in the paper.

Performance measures

The simulation results are presented in Table B2. From the analysis of the Table, it is evident that the number of removed trials is greater when the pairs of the means ages is “very different” and decreases when the pairs of the means age is “equal”. This demonstrates how the proposed procedure identifies and removes unbalanced trials. Furthermore, as expected, at low standard deviation values, with the difference between the means held constant, the number of removed trials increases. In contrast, with high standard deviation values, the number of removed trials decreases, as the probability of “overlap” between the means increases. For instance, in scenario 8, a number of trials between 0 and 4 were removed 89 times out of 1000 replications, and a number of trials between 5 and 9 were removed 462 times, and so on.

Table 2. Distribution of removed trials by scenarios.

No. of removed trials	Scenario 1	2	3	4	5	6	7	8	9
	(μ_{ctrl}, μ_{exp}) (50, 50)			(μ_{ctrl}, μ_{exp}) (55, 50)			(μ_{ctrl}, μ_{exp}) (60, 50)		
	$\sigma = 5$	$\sigma = 10$	$\sigma = 15$	$\sigma = 5$	$\sigma = 10$	$\sigma = 15$	$\sigma = 5$	$\sigma = 10$	$\sigma = 15$
0 – 4	951	965	964	100	656	825	0	89	454
5 – 9	48	35	36	461	301	164	2	462	432
10 – 14	1	0	0	355	42	10	78	364	106
15 – 19	0	0	0	82	1	1	568	81	7
20 – 24	0	0	0	2	0	0	352	4	1
Repetitions	1000	1000	1000	1000	1000	1000	1000	1000	1000
