# Supplementary material for: A statistical method for removing unbalanced trials with multiple covariates in meta-analysis
Source: PLoS One. 2023 Dec 15;18(12):e0295332. doi: 10.1371/journal.pone.0295332 (PMC10723740; doi:10.1371/journal.pone.0295332)
Supplement: S2 Appendix — (PDF) [file pone.0295332.s002.pdf]

S2 APPENDIX

The control meta-arm and the experimental meta-arm

Let S_i be the ith trial (for $i = 1, 2, \ldots, I$) of the original set of I trials, **S**, and n_i the number of enrolled patients. Let k be the number of arms ($k \geq 2$) of S_i. Generally, as in our case, $k = 2$, that is, the control arm ($ctrl_i$, for $k = 1$) and the experimental arm (exp_i, for $k = 2$), with number of patients n_{1i} and n_{2i}, respectively, and $n_{1i} + n_{2i} = n_i$.

Let ***ctrl*** = $\{ctrl_1, ctrl_2, \ldots, ctrl_I\}$ be the *control meta-arm*, i.e., the collection of all I control arms of **S**, each with ($n_{1I}, n_{12}, \ldots, n_{1I}$) patients, and $n_1 = \sum_{i=1}^{I} n_{1i}$ the total number of patients. Let ***exp*** = $\{exp_1, exp_2, \ldots, exp_I\}$ be the *experimental meta-arm*, i.e., the collection of all I experimental arms of **S**, each with ($n_{2I}, n_{22}, \ldots, n_{2I}$) patients, with $n_2 = \sum_{i=1}^{I} n_{2i}$ the total number of patients. Thus, $n = n_1 + n_2$ will be the number of patients enrolled in **S**.

The *empirical cumulative distribution function*

We build the Empirical Cumulative Distribution Function (ECDF) for a given PLV, for each meta-arm. Let the I elements of each meta-arm be a random sample drawn from a distribution with cumulative distribution function F. Let $\{\hat{\theta}_1, \hat{\theta}_2, \ldots, \hat{\theta}_I\}$ be the I ordered statistics of the arms for the PLV. Then, the ECDF will be defined as follows:

$$F_i(\theta) = \frac{\#\left(\hat{\theta}_i \leq \theta\right)}{n}, \quad -\infty < \theta < +\infty, \qquad \begin{cases} F_i(\theta) = 0, & \theta < \hat{\theta}_1 \quad i = 1 \\ F_i(\theta) = \frac{n_i}{n}, & \hat{\theta}_i < \theta < \hat{\theta}_{i+1}, \quad i = 1,, I-1 \\ F_i(\theta) = 1, & \hat{\theta}_I < \theta \quad i = I \end{cases} \tag{1}$$

where $\#\left(\widehat{\theta_i} \le \theta\right)$ is read the number of $\widehat{\theta}_i$'s less than or equal to θ, $n = \sum_{i=1}^{I} n_i$ is the total number of patients enrolled in the meta-arm, and n_i is the cumulative number of patients enrolled in all arms with a statistic $\widehat{\theta}_i$ less than or equal to θ. Therefore, the ratio (n_i/n) is the value of the ECDF at $\widehat{\theta}_i$. If all the arms have distinct $\widehat{\theta}_i$, then the number of jumps of $F_i(\theta)$ will be equal to I; otherwise, if there are two or more arms that share the same $\widehat{\theta}_i$, then the number of the jumps of $F_i(\theta)$ will be equal to the number of distinct $\widehat{\theta}_i$.

As an example, we reproduce below the table for the construction of the two ECDFs (see Fig. 4a) of the *ctrl* and exp meta-arms of the 11 trials of S1 of the Chol dataset, compared with the PLV mean(age).

Table 1. ECDFs for the *mean age* in the two meta-arms of S1 (*Chol* dataset).

**Trial label**	**Arm**	***mean*(*age*)** θ_i	**Pts** n_i	***ECDF*** $F_i(\theta)$
ALERT	Ctrl	50.0	1052	0.033
WOSCOPS	Ctrl	55.1	3293	0.135
SSSS	Ctrl	58.6	2223	0.204
GISSI-P	Ctrl	60.0	2133	0.270
LIPS	Ctrl	60.0	833	0.296
CARDS	Ctrl	61.8	1410	0.340
ASCOT-LLA	Ctrl	63.2	5137	0.500
HPS	Ctrl	65.2	10267	0.819
4D	Ctrl	65.7	636	0.838
GISSI-HF	Ctrl	68.0	2289	0.909
PROSPER	Ctrl	75.3	2913	1.000
Total (n_1)			32186	
ALERT	Exp	49.5	1050	0.033
WOSCOPS	Exp	56.3	3302	0.135
SSSS	Exp	58.6	2221	0.204
GISSI-P	Exp	59.7	2138	0.270
LIPS	Exp	60.0	844	0.297
CARDS	Exp	61.5	1428	0.341
ASCOT-LLA	Exp	63.1	5168	0.501
HPS	Exp	65.2	10269	0.820
4D	Exp	65.7	619	0.839
GISSI-HF	Exp	68.0	2285	0.910
PROSPER	Exp	75.4	2891	1.000
Total (n_2)			32215	
