# Supplementary material for: A statistical method for removing unbalanced trials with multiple covariates in meta-analysis
Source: PLoS One. 2023 Dec 15;18(12):e0295332. doi: 10.1371/journal.pone.0295332 (PMC10723740; doi:10.1371/journal.pone.0295332)
Supplement: S2 File — (PDF) [file pone.0295332.s004.pdf]

S2 File. The studies' selection procedure for the *Hep* dataset.

Id	Study name	Paper	Included in Brok et al.	Excluded (Reason)	Included	n
1	**Andreone1999a**	Andreone P, Cursaro C, Gramenzi A, Fiorino S, Di Giammarino L, Miniero R, et al. Interferon alpha plus ketoprofen or interferon alpha plus ribavirin in chronic hepatitis C non-responder to interferon alpha alone: results of a pilot study. *Italian Journal of Gastroenterology and Hepatology* 1999; **31**(8):688–94. [MEDLINE: 20192804].	**yes**		Yes	**1**
2	**Andreone1999a**	Andreone P, Gramenzi A, Cursaro C, di Giammarino L, Fiorino S, Scrivano P, et al. A randomized controlled trial of IFN alfa+ketoprofen or IFN alfa+ribavirin in HCV chronic active hepatitis (CAH) non-responder to IFN-alfa alone (abstract). Hepatology 1995;22(4 Pt 2):119A.	**no**		No	
3	**Andreone1999a**	Andreone P, Gramenzi A, Cursaro C, di Giammarino L, Fiorino S, Scrivano P, et al. IFN- alpha plus ketoprofen or ribavirin in HCV-positive chronic active hepatitis (CAH) non-responder to IFN-alpha alone: results of a randomized trial (abstract). Journal of Hepatology 1995;23 Suppl 1:94A.	**no**		No	
4	**Andreone1999b**	Andreone P, Cursaro C, Gramenzi A, di Giammarino L, Felline F, Miniero R, et al. A randomized controlled trial of leukocytic IFN-a vs. leukocytic IFN-a plus ribavirin in chronic hepatitis C resistant to a previous recombinant or lymphoblastoid IFN treatment. Italian Journal of Gastroenterology and Hepatology 1997;29(1):90.	**no**		No	
5	**Andreone1999b**	Andreone P, Cursaro C, Gramenzi A, Sbolli G, Fiorino S, di Giammarino L, et al . IFNalfa-n3 vs IFNalfa-n3 plus ribavirin in chronic hepatitis C (CHC) resistant to other IFNalfa treatments: results of a randomized multicenter trial (abstract). Hepatology 1997;26(4 Pt 2):216A.	**no**		No	
6	**Andreone1999b**	Andreone P, Gramenzi A, Cursaro C, Sbolli G, Fiorino S, di Giammarino L, et al. Interferon-alpha plus ribavirin in chronic hepatitis C resistant to previous interferon-alpha course: results of a randomized multicenter trial. Journal of Hepatology 1999;30(5):788–93. [MEDLINE: 99291925; :PMID: 10365803]	**yes**		Yes	**2**
7	**Ascione 1998**	Ascione A, de Luca M, Guardascione MA, Canestrini C, Galeota Lanza A, Astritto S, et al. Interferon plus ribavirin vs interferon alone in HCV chronic liver disease non responder to a previous cycle of interferon alone (abstract). Journal of Hepatology 1998;28 Suppl 1:198A.	**yes**	**1**	No	
8	**Barbaro 1998a**	Barbaro G, Di Lorenzo G, Soldini M, Giancaspro G, Bellomo G, Belloni G, et al. Interferon-alpha-2B and ribavirin in combination for chronic hepatitis C patients not responding to interferon-alpha alone: an Italian multicenter, randomized, controlled, clinical study. American Journal of Gastroenterology 1998;93(12):2445–51. [MEDLINE: 99075750; PMID: 9860407]	**yes**		Yes	**3**
9	**Barbaro 1998b**	Barbaro G, Belloni G, Ferrari L, Di Lorenzo G, Soldini M, Giancaspro G, et al. Alpha interferon 2b plus ribavirin in CHC relapser or not responder patients to alpha interferon 2b alone: an Italian multicenter randomized controlled clinical study (abstract). Hepatology 1998;28(4 Pt 2):476A.	**yes**	**1**	No	
10	**Barbaro 1999**	Barbaro G, Belloni G, Di Lorenzo G, Soldini M, Giancasprio G, Del Poggio P, et al. Alpha interferon 2b plus ribavirin in CHC relapser or not responder patients to alpha interferon 2b alone: an Italian multicenter randomized, controlled clinical study (abstract). Journal of Hepatology 1999;30 Suppl 1:116A.	**no**		No	
11	**Barbaro 1999**	Barbaro G, Di Lorenzo G, Belloni G, Ferrari L, Paiano A, Del Poggio P, et al. Interferon alpha-2B and ribavirin in combination for patients with chronic hepatitis C who failed to respond to, or relapsed after, interferon alpha therapy: a randomized trial. The American Journal of Medicine 1999;107(2):112–8. [MEDLINE: 99387796; PMID: 10460040]	**yes**		Yes	**4**
12	**Barbaro 2000a**	Barbaro G, Di Lorenzo G, Soldini M, Giancaspro G, Pellicelli A, Grisorio B, Barbarini G. The Ribavirin-Interferon in Chronic Hepatitis Italian Group Investigators. Evaluation of efficacy of interferon alpha-2b and ribavirin in combination in naive patients with chronic hepatitis C: an Italian multicenter experience. Journal of Hepatology 2000;33:448–55.	**yes**		Yes	**5**

**13**	**Bell 1999**	Bell H, Hellum K, Harthug S, Myrvang B, Ritland S, Maeland A, et al.Treatment with interferon-alpha2a alone or interferon-alpha2a plus ribavirin in patients with chronic hepatitis C previously treated with interferon-alpha2a. Scandinavian Journal of Gastroenterology 1999;34(2):194–8. [MEDLINE: 99401091; PMID: 10470088]	**yes**		Yes	**6**
**14**	**Bellobuono 1997**	Bellobuono A, Mondazzi L, Tempini S, Silini E, Vicari F, Idéo G. Ribavirin and interferon-alpha combination therapy vs interferon-alpha alone in the retreatment of chronic hepatitis C: a randomized clinical trial. Journal of Viral Hepatitis 1997;4(3):185–91. [MEDLINE: 97325474; PMID: 9181527]	**yes**		Yes	**7**
**15**	**Bellobuono 1997**	Bellobuono A, Tempini S,Mondazzi L, Idéo G. Retreatment of chronic hepatitis C with ribavirin and alpha interferon: a randomized controlled study in non-responder or relapse patients (abstract). Journal of Hepatology 1996;25 Suppl 1: 82A	**no**		No	
**16**	**Bellobuono 1999**	Bellobuono A, Tempini S, Mondazzi L, Brasca P, Marino F, Ideo G. Twelve month retreatment with IFNand ribavirin or IFN alone in relapser patients with chronic hepatitis C (abstract). Hepatology 1999;30(4 Pt 2):263A.	**yes**	**1**	No	
**17**	**Bellobuono 2000a**	Bellobbuono A, Mondazzi L, Tempini S, Chiodo F, Magliano E, Furione L, Idéo G. Early addition of ribavirin to interferon in chronic hepatitis C not responsive to interferon monotherapy. Journal of Hepatology 2000;33: 463–8.	**yes**		Yes	**8**
**18**	**Bellobuono 2000a**	Bellobuono A, Tempini S, Mondazzi L, Di Napoli M, Ideo G. Significant improvement of virological response after ribavirin addition to IFN in still viremic patients after the initial month of IFN therapy for chronic hepatitis C (abstract). Journal of Hepatology 1998;28 Suppl 1:112.	**no**		No	
**19**	**Bellobuono 2000a**	Bellobuono A, Tempini S, Mondazzi L, Idèo G. Effiacy of ribavirin addition to alpha IFN after the initial month of therapy in unresponsive patients: relationship with HCV RNA titre and HCV genotype (abstract). Journal of Hepatology 1999;30(Suppl 1):115.	**no**		No	
**20**	**Berg 2000a**	Berg T, Hoffmann RM, Teuber G, Leifeld L, Lafrenz M, Baumgarten R, et al. Efficacy of a short-term ribavirin plus interferon alfa combination therapy followed by interferon alfa alone in previously untreated patients with chronic hepatitis C: a randomized multicenter trial. Liver 2000;20 (6):427–36.	**yes**		Yes	**9**
**21**	**Berg 2000a**	Berg T, Hoffmann RM, Teuber G, Leifeld L, Lafrenz M, Baumgarten R, et al. Efficacy of short-term induction therapy with ribavirin plus interferon alfa in previously untreated patients with chronic hepatitis C (abstract). Journal of Hepatology 1999;30(Suppl 1):70.	**no**		No	
**22**	**Berg 2000a**	Berg T, Hoffmann RM, Teuber G, Leifeld L, Lawrenz M, Baumgarten R, et al. Efficacy of short-term ribavirin plus interferon alfa combination therapy followed by interferon alfa alone in previously untreated patients with chronic hepatitis C. A randomized trial (abstract). Hepatology 1998; 28(4):373A.	**no**		No	
**23**	**Berg 2000a**	Hoffmann RM, Berg T, Teuber G, Prummer OLL, Jung MC, Spengler U, et al. Interferon-antibodies and the breakthrough phenomenon during ribavirin/interferon-alpha combination therapy and interferon-alpha monotherapy of patients with chronic hepatitis C. Zeitschrift für Gastroenterologie 1999;37(8):715–23. [MEDLINE: 99424393; PMID: 10494606]	**no**		No	
**24**	**Berg 2000a**	Hoffmann RM, Jung MC, Motz R, Gossl C, Emslander HP, Zachoval R, et al. Sarcoidosis associated with interferonalpha therapy for chronic hepatitis C. Journal of Hepatology 1998;28(6):1058–63. [MEDLINE: 98335905; PMID: 9672184]	**no**		No	
**25**	**Berg 2000a**	Zeuzem S, Schmidt JM, Lee JH, von-Wagner M, Teuber G, Roth WK. Hepatitis C virus dynamics in vivo: effect of ribavirin and interferon alfa on viral turnover. Hepatology 1998;28(1):245–52. [MEDLINE: 98319201; PMID: 9657119]	**no**		No	
**26**	**Berg 2000b**	Berg T, Kaul U, Maumann, Wiedemann B, Hopf U. Influence of ribavirin on the dynamics of hepatitis C viremia in Interferon-alpha-treated Patients with response or nonresponse [Einfluss von ribavirin auf die Dynamik der hepatitis–C–virämie bei interferon–alpha–behandelten patienten mit response oder nonresponse]. Zeitschrift für Gastroenterologie 2000;38:881–6.	**yes**	**5**	No	
**27**	**Berg 2000b**	Berg T, Naumann U, Wiedenmann B, Hopf U. Kinetics of hepatitis C viremia in interferon plus ribavirin treated patients (abstract). Hepatology 1998;28(4 Pt 2):373A.	**no**		No	

**28**	**Boucher 2003**	Boucher EJ, Jacquelinet S, Canva V, Turlin B, Colimon R, Jacquelint C, et al. High rate long term response after one year interferon plus ribavirin treatment for hepatitis C relapsers. Results of a controlled randomised study in 191 patients (abstract). Journal of Hepatology 2001;34 Suppl 1: 167.	**no**		No	
**29**	**Boucher 2003**	Boucher EJ, Jacquelinet S, Canva V, Turlin B, Jacquelinet C, Colimon R, et al. High rate of long-term virological response after a 1-year course of interferon +/- ribavirin in chronic hepatitis C relapsers. Results of a 191 patients randomized trial. Liver International 2003;23:255–61	**yes**		Yes	**10**
**30**	**Bresci 2000**	Bresci G, Parisi G, bertoni M, Capria A. High-dose interferon plus ribavirin in chronic hepatitis C not responding to recombinant alpha-interferon. Digestive and Liver Disease 2000;32:703–7.	**yes**		Yes	**11**
**31**	**Bresci 2000**	Bresci G, Parisi G, Bertoni M, Scatena F, Capria A. Interferon plus ribavirin in chronic hepatitis C nonresponders to recombinant alpha-interferon. Journal of Viral Hepatitis 2000;7(1):75–81.	**no**		No	
**32**	**Brillanti 1995**	Brillanti S, Foli M, Masci C, Miglioli M. Three-year follow-up of chronic hepatitis C patients treated with ribavirin plus interferon-alpha combination therapy:	**no**		No	
**33**	**Brillanti 1995**	Brillanti S, Garson J, Foli M, Whitby K, Deaville R, Masci C, et al.A pilot study of combination therapy with ribavirin plus interferon alfa for interferon alfa-resistant chronic hepatitis C. Gastroenterology 1994;107(3):812–7. [MEDLINE: 94357381; : PMID: 7521308]	**yes**		Yes	**12**
**34**	**Brillanti 1995**	Brillanti S, Masci C, Miglioli M, Barbara L. Ribavirin combined with alpha interferon treatment for chronic HCV infection resistant to alpha interferon therapy (abstract). Journal of Hepatology 1993;18 Suppl 1:101.	**no**		No	
**35**	**Brillanti 1995**	Brillanti S, Miglioli M, Barbara L. Combination antiviral therapy with ribavirin and interferon alfa in interferon alfa relapsers and non-responders: Italian experience. Journal of Hepatology 1995;23 Suppl 2:13–5. [MEDLINE:96351138; : PMID: 8720288]	**no**		No	
**36**	**Brouwer 2004**	Brouwer JT, Hansen BE, Schalm SW. Low relapse rate in chronic hepatitis C treated with 18 months interferon-alpha and ribavirin as compared to 6 months combination therapy and to 18 months monotherapy. A Benelux study in 300 patients (abstract). Hepatology 2000;32(4 pt 2):307A.	**no**		No	
**37**	**Brouwer 2004**	Brouwer JT, Nevens F, Bekkering FC, Bourgeois N, Van Vlierberghe H, Weegink CJ, et al.Reduction of relapse rates by 18-month treatment in chronic hepatitis C. A Benelux randomized trial in 300 patients. Journal of Hepatology 2004;40(4):689–95.	**yes**		Yes	**13**
**38**	**Brouwer 2004**	Brouwer JT, Schalm SW. Reduction of relapse in chronic HCV: a Benelux study in 300 patients. Journal of Hepatology 2001;34(Suppl 1):16.	**no**		No	
**39**	**Brouwer 2004**	Veldt BJ, Brouwer JT, Adler M, Nevens F, Michielsen P, Delwaide J, et al. Retreatment of hepatitis C non-responsive to interferon. A placebo controlled randomized trial of ribavirin monotherapy versus combination therapy with Ribavirin and Interferon in 121 patients in the Benelux. BMC Gastroenterolology 2003;29;3(1):24.	**no**		No	
**40**	**Bugliescu 2000**	Bugliescu L, Cojocaru L, Micu L, Copaci I. Results of associated treatment with interferon (IFN) + ribavirin in HCV active chronic hepatitis (abstract). Journal of Gastroenterology and Hepatology 2000;15 Suppl:F83.	**yes**	**1**	No	
**41**	**Caremani 1996**	Caremani M, Benci A, Castellacci R, Tacconi D. A randomised controlled trial of leukocyte IFNalpha+ribavirin in HCV chronic hepatitis relapsing patients (abstract). Hepatology 1996;24(4 Pt 2):395A. [:CN–00186171]	**yes**	**1**	No	

42	**Cavaletto 2000**	Cavalletto L, Chemello L, Donada C, Casarin P, Belussi F, Bernardinello E, et al. The pattern of response to interferon alpha (alpha-IFN) predicts sustained response to a 6-month alpha-IFN and ribavirin retreatment for chronic hepatitis C. TVVH Study Group. Journal of Hepatology 2000;33(1):128–34. [MEDLINE: PMID: 10905596, UI: 20361531]	**yes**	**2**	No	
43	**Cavaletto 2000**	Chemello L, Cavalletto L, Bernardinello E, Donada C, Belussi F, Casarin P, et al. Retreatment of chronic hepatitis C (CHC) with sequential interferon-ribavirin combination (IFN-RIBA) therapy (abstract). Journal of Hepatology 1998; 28 Suppl 1:109.	**no**		No	
44	**Chapman 2001**	Chapman BA, Stace NH, Edgar CL, Bartlett SE, Frampton CMA, Scahill SL, et al.Interferon-alpha 2a/Ribavirin versus interferon-alpha 2a alone for the retreatment of hepatitis C patients who relapse after standard course of interferon. The New Zealand Medical Journal 2001;114(1128):103–4.	**yes**	**6**	no	
45	**Chemello 1995**	Chemello L, Cavalletto L, Bernardinello E, Guido M, Pontisso P, Alberti A. The effect of interferon alfa and ribavirin combination therapy in naive patients with chronic hepatitis C. Journal of Hepatology 1995;23 Suppl 2:8–12. [MEDLINE: 96351137; : PMID: 8720287]	**yes**		Yes	**14**
46	**Chemello 1995**	Chemello L, Cavalletto L, Bernardinello E, Silvestri E, Benvegnu L, Pontisso P, et al. Response to ribavirin, to interferon and to a combination of both in patients with chronic hepatitis C and its relation to HCV genotypes (abstract). Journal of Hepatology 1994;21 Suppl 1:12.	**no**		No	
47	**Cheng 2002**	Cheng PN, Chow NH, Hu SC Young KC, Chen CY, Jen CM, et al. Clinical comparison of high-dose interferonalpha2b with or without ribavirin for treatment of interferon-relapsed chronic hepatitis C. Digestive and Liver Disease 2002;34:851–6.	**yes**	**6**	No	
48	**Davis 1998**	Davis GL, Esteban-Mur R, Rustgi V, Hoefs J, Gordon S, Trepo C, et al. Retreatment of relapse after interferon therapy for chronic hepatitis C: an international randomized controlled trial of interferon plus ribavirin vs interferon alone (abstract). Hepatology 1997;26(4 Pt 2):274A.	**no**		No	
49	**Devis 1998**	Davis GL, Esteban-Mur R, Rustgi V, Hoefs J, Gordon SC, Trepo C, et al. Interferon alfa-2b alone or in combination with ribavirin for the treatment of relapse of chronic hepatitis C. New England Journal of Medicine 1998;339(21): 1493–9. [MEDLINE: UI: 99025347; : PMID: 9819447]	**yes**		Yes	**15**
50	**Devis 1998**	Neary MP, Cort S, Bayliss MS, Ware JE Jr. Sustained virologic response is associated with improved health-related quality of life in relapsed chronic hepatitis C patients. Seminars in Liver Disease. 1999;19 Suppl 1:77–85. [MEDLINE: 99279312; : PMID: 10349695]	**no**		No	
51	**Devis 1998**	Ware JE Jr, Bayliss MS, Mannocchia M, Davis GL, the International Hepatitis Interventional Therapy Group. Health-related quality of life in chronic hepatitis C: impact of disease and treatment response. Hepatology 1999;30(2): 550–5. [MEDLINE: 99350396; : PMID: 10421667]	**no**		No	
52	**Dettmer 2002**	Dettmer R, Reinus JF, Clain DJ, Aytaman A, Levendoglu H, Bloom AA, et al. Interferon-alpha-2b for retreatment of chronic hepatitis C. Hepatogastroenterology 2002;49:758–63.	**yes**		Yes	**16**
53	**Dettmer 2002**	Dettmer RM, Aytaman A, Bloom AA, Clain DJ, Magun A, Meyer D, et al. Multicenter placebo-controlled, doubleblind trial of interferon alpha-2b plus ribavirin vs. interferon alone for retreatment of non-responders and relapsers with chronic hepatitis C who have failed previous treatment with interferon: a 48 week review (abstract). Hepatology 1999;30 (4 Pt 2):201A.	**no**		No	
54	**Dettmer 2002**	Sarabanchong V, Reinus J, Clain DJ, Aytaman A, Levendoglu H, Bloom AA, et al. Double-blind placebocontrolled trial of interferon alpha-2b plus ribavirin versus interferon alpha-2b for retreatment of patients with chronic hepatitis C who failed previous treatment with interferon alpha (abstract). Hepatology 1998;28(Suppl 4):703A.	**no**		No	
55	**el-Zayadi 1999**	el-Zayadi A, Selim O, Hadda S, Simmmonds C, Hamdy H, Badran HM, et al.Combination treatment of interferon alpha-2b and ribavirin in comparison to interferon monotherapy in treatment of chronic hepatitis C genotype 4 patients. Italian Journal of Gastroenterology and Hepatology 1999;31(6):472–5.	**yes**	**6**	No	
56	**Ferenci 2001**	Ferenci P, Stauber R, Hackl W, Datz C, Gschwantler W, Steindl P, et al. A prospective, randomized controlled trial of high dose interferon-alfa plus ribavirin in interferonnonresponders with chronic hepatitis C (abstract). Hepatology 1997;26(4 Pt 2):415A.	**no**		No	

57	Ferenci 2001	Ferenci P, Stauber R, Steindl-Munda P, Gschwantler M, Fickert P, Datz C, et al. Interim analysis of a randomized controlled trial of combination of ribavirin and high dose interferon-alfa in interferon nonresponders with chronic hepatitis C. Journal of Viral Hepatitis 1999;1 Suppl 1:53–8. [MEDLINE: 20223856; : PMID: 10760037]	no		No	
58	Ferenci 2001	Ferenci P, Stauber R, Steindl-Mundi P, Gschwantler M, Fickert P, Datz C, et al. Treatment of patients with chronic hepatitis C not responding to interferon with high-dose interferon alpha with or without ribavirin: final results of a prospective randomised trial. European Journal of Gastroenterology and Hepatology 2001;13(6):699–705.	yes		Yes	17
59	Fried 2002	Fried MW, Hoots K, Peter J, Gaglio PJ, Talbut D, Davis C, et al. Hepatitis in adults and adolescents with hemophilia: a randomized, controlled trial of interferon alfa-2b and ribavirin. Hepatology 2002;36(4 Pt 1):967–72.	yes	4	No	
60	Fried 2002b	Bosques-Padilla F, Trejo-Estrada R, Campollo-Rivas O, Cortez-Hernandez C, Dehesa-Violante M, Maldonado-Garza H, er al. Peginterferon alfa-2a plus ribavirin for treating chronic hepatitis C virus infection: analysis of Mexican patients included in a multicenter international clinical trial. Annals of Hepatology 2003;2(3):135–9.	no		No	
61	Fried2002b	Fried MW, Shiffman ML, Reddy KR, Smith C, Marinos G, Goncales F Jr, et al. Peginterferon alfa-2a plus ribavirin for chronic hepatitis C viral infection. New England Journal of Medicine 2002;347(13):975–82.	yes		Yes	18
62	Fried 2002b	Hassanein T, Cooksley G, Sulkowski M, Smith C, Marinos G, Lai MY, et al. The impact of peginterferon alfa-2a plus ribavirin combination therapy on health-related quality of life in chronic hepatitis C. Journal of Hepatology 2004;40(4):675–81.	no		No	
63	Gerotto 1999	Gerotto M, Sullivan DG, Polyak SJ, Chemello L, Cavalletto L, Pontisso P, et al. Effect of retreatment with interferon alone or interferon plus ribavirin on hepatitis C virus quasispecies diversification in nonresponder patients with chronic hepatitis C. Journal of Virology 1999;73(9):7241–7.	yes	6	No	
64	Glue 2000	Glue P, Rouzier-Panis R, Raffanel C, Sabo R, Gupta SK, Salfi M, et al. A dose-ranging study of pegylated interferon alfa-2b and ribavirin in chronic hepatitis C. Hepatology 2000;32(3):647–53.	yes	3	No	
65	Gross 1999a	Gross JB, Lindor KD, Abdelmalek MF, Poterucha JJ, Brandhagen DJ, Czaja AJ, et al. Interferon alpha 2b 5MU tiw with or without ribavirin as initial treatment for hepatitis C (abstract). Hepatology 1999;30(4 Pt 2):634A.	yes	1	No	
66	Gross 1999b	Gross JB, Lindor KD, Abdelmalek MF, Poterucha JJ, Brandhagen DJ, Czaja AJ, et al. Interferon alpha 2b 5MIU tiw,+/- 4-week daily interferon induction, +/- ribavirin, for re-treatment of interferon non-responders with chronic hepatitis C (abstract). Hepatology 1999;30(4 Pt 2):634A.	yes	1	No	
67	Gross 1999b	Peine CG, Albrecht JK, Roel JP, Gundersen BA, Kirchner JP, Zins B, et al. A comparison of standard and induction interferon therapy with and without initial ribavirin in treatment naive patients with chronic hepatitis C (abstract). Hepatology 2000;32((4 Pt 2)):364A.	no		No	
68	Khakoo 1998	Khakoo S, Glue P, Grellier L, Wells B, Bell A, Dash C, et al. Ribavirin and interferon alfa-2b in chronic hepatitis C: assessment of possible pharmacokinetic and pharmacodynamic interactions. British Journal of Clinical Pharmacology 1998;46(6):563–70. [MEDLINE: 99077061; : PMID: 9862245]	yes	2	No	
69	Koshy 2000	Koshy A, Marcellin P, Martinot M, Madda JP. Improved response to ribavirin interferon combination compared with interferon alone in patients with type 4 chronic hepatitis C without cirrhosis. Liver 2000;20(4):335–9.	yes	6	No	
70	Lai 1996	Lai MY, Kao JH, Yang PM, Wang JT, Chen PJ, Chan KW, et al. Long-term efficacy of ribavirin plus interferon alfa in the treatment of chronic hepatitis C. Gastroenterology 1996;111(5):1307–12. [MEDLINE: 97054348; : PMID: 8898645]	yes	6	No	
71	Lai 1996	Lai MY, Yang PM, Kao JH, Wang JT, Lee HS, Chen DS. Combination therapy of alfa-interferon and ribavirin in patients with chronic hepatitis C: an interim report (abstract). Hepatology 1993;18(4 Pt 2):93A.	no		No	

72	**Lédinghen 2002a**	Lédinghen V, Trimoulet P,WinnockM, Foucher J, Bourliérec M, Desmorat H, et al. Daily or three times a week interferon alfa-2b in combination with ribavirin or interferon alone for the treatment of patients with chronic hepatitis. Journal of Hepatatology 2002;36:672–80.	**yes**		Yes	**19**
73	**<u>Lédinghen 2002b</u>**	De Lédinghen V, Bernard PH, Trimoulet P, Bourlière M, Portal I, Rémy AJ, et al. Retreatment for chronic hepatitis C interferon non-responsders patients. Preliminary results of a multicenter randomised controlled trial of interferon plus ribavirin versus interferon alone (abstract). Hepatology 1998;28((4 Pt 2)):374A.	**no**		No	
74	**Lédinghen 2002b**	Lédinghen VL, Trimoulet P, Bernard PH, Bourliere M, Portal I, Rémy AJ, et al. Daily or three times per week interferon alpha-2b combination with ribavirin or interferon alone for the treatment of patients with chronic hepatitis C not responding to previous interferon alone. Journal of Hepatology 2002;36(6):819–26.	**yes**		Yes	**20**
75	**Malik 2002**	Malik AH, Kumar KS, Malet PF, Ostapowicz G, Adams G, Wood M, et al. A randomised trial of high-dose interferon alpha-2b, with or without ribavirin, in chronic hepatitis C patients who have not responded to standard dose interferon. Alimentary Pharmacology and Therapeutics 2002; 16(3):381–8.	**yes**		Yes	**21**
76	**<u>Mangia 2001</u>**	Mangia A, Villani MR, Minerva N, Carretta V, Vinelli F, Romano G, et al. End of therapy response in naive pts: 6MU IFN alpha 2B+ribavirin vs IFN alpha 2B 6MU alone (abstract). Journal of Hepatology 1999;30 Suppl 1:137.	**no**		No	
77	**Mangia 2001**	Mangia A, Villani MR, Minerva N, Leandro G, Bacca D, Cela M, et al. Efficacy of 5MU of interferon in combination with ribavirin for naive patients with chronic hepatitis C virus: a randomised controlled trial. Journal of Hepatology 2001;34:441–6.	**yes**		Yes	**22**
78	**Marcellin 1999**	Marcellin P, Hezode C, Castelnau C, Barange K, Couzigou P, Larrey D, et al. Randomized controlled trial of combination therapy with interferon (IFN) alfa-2A and ribavirin, in patients with chronic hepatitis C who relapsed after interferon therapy (abstract). Hepatology 1999;30(4 Pt 2):192A.	**yes**	**1**	No	
79	**<u>Di Marco 2002</u>**	Marco DV, Ferraro D, Almasio PL, Vaccaro A, Parisi P, Capello M. Early Viral clearance and sustained response in chronic Hepatitis C: The effect of adding ribavirin after high dose IFN induction (abstract). Hepatology 2001 2001; 4 pt 2:333A.	**no**		No	
80	**Di Marco 2002**	Marco DV, Ferraro D, Almasio P, Vaccaro A, Parisi P, Cappello M, et al. Early viral clearance and sustained response in chronic hepattis C: a controlled trial of interferon and ribavirin after high-dose interferon induction. Journal of Viral Hepatitis 2002;9(2):453–9.	**yes**		Yes	**23**
81	**<u>McHutchison 1998</u>**	McHutchison JG, Gordon S, Schiff E, Shiffman M, Lee W, Rustgi V, et al. Interferon alfa 2B alone or in combination with ribavirin in naive chronic HCV patients: a US multicenter trial (abstract). Hepatology 1998;28(4 Pt 2):387A.	**no**		No	
82	**<u>McHutchison 1998</u>**	McHutchison JG, Gordon SC, Morgan T, Ling MH, Gaurad JJ, Albrect J, et al. Predicting response to initial therapy with interferon alfa during ribavirin in chronic hepatitis C using serum HCV RNA during therapy (abstract). Hepatology 1999;30(4 Pt 2):365A.	**no**		No	
83	**McHutchison 1998**	McHutchison JG, Gordon SC, Schiff ER, Shiffman ML, Lee WM, Rustgi VK, et al.Interferon alfa-2b alone or in combination with ribavirin as initial treatment for chronic hepatitis C. New England Journal of Medicine 1998;339(21): 1485–92. [MEDLINE: 99025346; : PMID: 9819446]	**yes**		Yes	**24**
84	**<u>McHutchison 1998</u>**	McHutchison JG, Ware JE, Bayliss MS, Pianko S, Albrecht JK, Cort S, et al. The effects of interferon alpha-2b in combination with ribavirin on health related quality of life and work productivity. Journal of Hepatology 2001;34: 140–7.	**no**		No	
85	**<u>McHutchison 1998</u>**	Neumann AU, Dahari H, Conrad A, Pianko S, McHutchison JG. Early prediction and mechanism of the ribavirin/IFN-aloha dual therapy effect on chronic hepatitis C virus (HCV) infection (abstract). Hepatology 1999;30(4Pt 2):309A.	**no**		No	
86	**<u>McHutchison 1998</u>**	Ware J, Bayliss M, McHutchison JG, Pianko S, Cort S, Albrecht J, et al. The impact of therapy with interferon alfa 2B plus ribavirin on health related quality of life and work productivity in chronic hepatitis C patients (abstract). Hepatology 1999;30(4 Pt 2):193A.	**no**		No	

**87**	**<u>Milella 1999</u>**	Milella M, Santantonio T, Pietromatera G, Maselli R, Casalino C, Appice A, et al. Ribavirin plus IFN vs IFN alone in the treatment of either non-responder or relapser patients with chronic hepatitis C (abstract). Journal of Hepatology 1997;26 Suppl 1:181.	**no**		No	
**88**	**Milella 1999**	Milella M, Santantonio T, Pietromatera G, Maselli R, Casalino C, Mariano N, et al. Retreatment of nonresponder or relapser chronic hepatitis C patients with interferon plus ribavirin vs interferon alone. Italian Journal of Gastroenterology and Hepatology 1999;31(3):211–5.	**yes**		Yes	**25**
**89**	**Nunes 1999**	Nunes DP, Anastopoulos H, Gordon F, Chopra S, Petruff C, et al. Double-blind placebo controlled study of interferon versus interferon plaus ribavirin for the treatment of hepatitis C in patients who previously failed interferon monotherapy (abstract). Hepatology 1999;30(4 Pt 2):199A.	**yes**	**1**	No	
**90**	**Pawlotsky 1998**	Castéra L, Germanidis G, Frainais PO, Hézole C, Dhumeaux D, Pawlotsky JM. Early changes in HCV hypervariable region 1 (HVR1) and non structural (NS) 5A gene quasispecies during interferon (IFN)-alpha or ribavirin treatment: clues to the mechanisms of HCV resistance to antiviral therapy (abstract). Hepatology 1998;28(4 Pt 2):288A.	**yes**	**1**	No	
**91**	**Pawlotsky 1998**	Pawlotsky JM, Dahan H, Conrad A, Lonjon I, Hézole C, Germanidis G, et al. Effect of intermittent interferon (IFN), daily IFN and IFN plus ribavirin induction therapy on hepatitis C virus (HCV) genotype 1b replication kinetics and clearance (abstract). Hepatology 1998;28(4 Pt 2):288A.	**no**		No	
**92**	**Piccolo 2004**	Piccolo P, Horst BK, Angelico F, Gentile S, Francioso S, Tarquini P, et al. Peg-interferon alpha-2a monotherapy vs Peg-interferon alpha-2a and ribavirin in naive patients with chronic hepatitis C: preliminary results of the SMIEC 2 trial in patients with early viral clearance on Peg-interferon monotherapy (abstract). Journal of Hepatology 2004;40 (Suppl 1):148.	**yes**	**1**	No	
**93**	**<u>Pockros 2003</u>**	Pockros PJ, Jolla L. A multi-center randomised trial to determine the safety and tolerability of combined treatment with ribavirin and infergen for HCV in naive patients (abstract). Journal of Hepatology 2001;34 Suppl 1:179.	**no**		No	
**94**	**Pockros 2003**	Pockros PJ, Reindollar R, McHutchinson J, Reddy R, Wright T, Boyd DG, et al. The safety and tolerability of daily infergen plus ribavirin in the treatment of naive chronic hepatitis C patients. Journal of Viral Hepatitis 2003;10(1):55–60.	**yes**		Yes	**26**
**95**	**<u>Pol 1999</u>**	French Multicenter Study Group, Pol S, Berthelot P, Brechot C. Ribavirin-interferon vs interferon (2b-IFN) alone in non-responders to IFN in chronic hepatitis C (abstract). Hepatology 1996;24(4 Pt 2):356A.	**no**		No	
**96**	**Pol1999**	Pol S, Couzigou P, Bourlière M, Abergel A, Combis JM, Larrey D, et al. A randomized trial of ribavirin and interferon-alpha vs. interferon-alpha alone in patients with chronic hepatitis C who were non-responders to a previous treatment. Multicenter Study Group under the coordination of the Necker Hospital, Paris, France. Journal of Hepatology 1999;31(1):1–7. [MEDLINE: 99351563; PMID: 10424277]	**yes**		Yes	**27**
**97**	**Pol 2000a**	Pol S, Nalpas B, Bourlière M, Couzigou P, Tran A, Abergel A, et al. Combination of ribavirin and interferonalfa surpasses high doses of interferon-alfa alone in patients with genotype 1b-related chronic hepatitis. Hepatology 2000;31(6):1338–44. [MEDLINE: 20287465; : PMID:10827161]	**yes**		Yes	**28**
**98**	**<u>Portal 2003</u>**	Portal-Bartolomei I, Bourlière M, Halfon P, De Lédinghen V, Bernard P, Botti G, et al. Retreatment with interferon ribavirine according to viremia of interferon responder relapser patients: preliminary results of a prospective French multicenter randomized controlled study (abstract). Hepatology 1999;30(4 Pt 2):194A.	**no**		No	
**99**	**Portal 2003**	Portal I, Bourliere M, Halfon P, De Ledinghen V, Couzigou P, Bernard PH, Blanc F, et al. Retreatment with interferon and ribavirin vs interferon alone according to viraemia in interferon responder-relapser hepatitis C patients: a prospective multicentre randomized controlled study. Journal of Viral Hepatitis 2003;10(3):215–23.	**yes**		Yes	**29**
**100**	**<u>Portal 2003</u>**	Portal I, Bourlière M, Halfon P, de Leidinghen V, Couzigou P, Bernard PH, et al. Retreatment with interferon-ribavirine according to viremia of interferon responders-relapser patients; French multicenter randomized controlled trial. Journal of Hepatology 2000;32 Suppl 2:97.	**no**		No	

**102**	**Poynard 1998a**	Neuman MG, Benhamou JP, Shear NH, Martinot M, Malkiewicz J, Boyer N, et al. Interleukine 6 level normalization during interferon-ribavirin combination therapy in chronic hepatitis C (abstract). Journal of Hepatology 1999;30 Suppl 1:242.	**no**		No	
**102**	**Poynard 1998a**	Neuman MG, Benhamou JP, Shear NH, Martinot M, Malkiewicz J, Boyer N, et al. Serum tumor necrosis factor as predictor of sustained response to alpha interferon-ribavirin combination therapy in chronic hepatitis C (abstract). Journal of Hepatology 1999;30 Suppl 1:257.	**no**		No	
**103**	**Poynard 1998a**	Neuman MG, Shear NH, Benhamou JP, Malkiewitz I, Katz GG, Boyer N, et al. Serum cytokine levels in patients with chronic hepatitis C are predictive value for the study outcome (abstract). Hepatology 1999;30(4 Pt 2):595A.	**no**		No	
**104**	**Poynard 1998a**	Poynard T, Marcellin P, Lee S, Niederau, Minuk G, Ideo G, et al. An international randomized trial of interferon alfa-2B and ribavirin (intron A/rebetol) 48 or 24 weeks vs intron a 48 weeks for first line treatment of chronic hepatitis C (abstract). Hepatology 1998;28(4 Pt 2):387A.	**no**		No	
**105**	**Poynard 1998a**	Poynard T, Marcellin P, Lee SS, Niederau C, Minuk GS, Ideo G, et al. Randomised trial of interferon alpha2b plus ribavirin for 48 weeks or for 24 weeks versus interferon alpha2b plus placebo for 48 weeks for treatment of chronic infection with hepatitis C virus. Lancet 1998;352(9138):1426–32. [MEDLINE: 99023142; : PMID: 9807989]	**yes**		Yes	**30**
**106**	**Poynard 1998a**	Rasul I, Liu M, Ning Q, Heathcote J, Levy G. Treatment of hepatitis C infection with interferon +/- ribavirin promoted conversion of TH2 to TH1 cytokine profile and decreased inflammation (abstract). Hepatology 1999;30(4 pt 2):594A.	**no**		No	
**107**	**Reichard 1998**	Reichard O, Norkrans G, Fryden A, Braconier JH, Alaeus A, Glaumann H, et al. Interferon-alpha and ribavirin versus interferon-alpha alone as therapy for chronic hepatitis C - a randomized double-blind placebo-controlled study (abstract). Hepatology 1996;24(4 Pt 2):356A.	**no**		No	
**108**	**Reichard 1998**	Reichard O, Norkrans G, Fryden A, Braconier JH, Sonnerborg A, Weiland O. Comparison of 3 quantitative HCV RNA assays-accuracy of baseline viral load to predict treatment outcome in chronic hepatitis C. Scandinavian Journal of Infectious Diseases 1998;30(5):441–6.	**no**		No	
**109**	**Reichard 1998**	Reichard O, Norkrans G, Fryden A, Braconier JH, Sonnerborg A, Weiland O. Randomised, double-blind, placebo-controlled trial of interferon alpha-2b with and without ribavirin for chronic hepatitis C. Lancet 1998;351 (9096):83–7. [MEDLINE: 98102664; : PMID: 9439491]	**yes**		Yes	**31**
**110**	**Ricchiuti 1996**	Ciccorossi CP, Ricchiuti A, Petruccelli S, Costa F, Bellini M, Arpe P, et al. Antiviral effect of ribavirin plus interferon in chronic hepatitis C (abstract). Hepatology 1998;28(4 Pt 2):474A.	**yes**	**1**	No	
**111**	**Ricchiuti 1996**	Ricchiuti A, Ciccorossi P, Costa F, Bellini M, Tumino E, Petruccelli S, et al. Combination therapy with interferon alpha and ribavirin in interferon alpha relapsers (abstract). Hepatology 1996;24(4 Pt 2):395A.	**no**		No	
**112**	**Ricchiuti 1999**	Ricchiuti A, Ciccorossi P, Costa F, Bellini M, Da Massa Carara P, Arpe P, et al. Daily administration of interferon alpha 2b and utility of combination therapy with ribavirin as initial treatment for chronic hepatitis C (abstract). Hepatology 1999;30(4 Pt 2):627A.	**yes**	**1**	No	
**113**	**Ricchiuti 1999**	Ricchiutti PC, Costa F, Bellini M, Carrara PDM, Arpe P, Petruccelli S, et al. Daily administration of intervention alpha 2b and utility of combination therapy with ribavirin vs. initial treatment for chronic hepatitis C: follow up of patients (abstract). Hepatology 2000;32((4 Pt 2)):366A.	**no**		No	
**114**	**Salmeron 1999**	Salmeron J, Perez-Ruiz M, Ruiz-Extremera A, Torres C, Lavin I, Quintero D, et al. Interferon versus ribavirin plus interferon in chronic hepatitis C previously resistant to interferon: a randomized trial (abstract). Hepatology 1996; Vol. 24, issue 4 Pt 2:395A.	**no**		No	
**115**	**Salmeron1999**	Salmeron J, Ruiz-Extremera A, Torres C, Rodriguez-Ramos L, Lavin I, Quintero D, et al. Interferon versus ribavirin plus interferon in chronic hepatitis C previously resistant to interferon: a randomized trial. Liver 1999;19(4):275–80. [MEDLINE: 99387380; : PMID: 10459624]	**yes**		Yes	**32**
**116**	**Salmeron 2003**	Salmeron J, Diago M, Andrada R, Perez R, Sola R, Romero M, et al. Interferon alpha-2a in combination therapies for the treatment of chronic hepatitis C in prior non-responders to interferon monotherapy (abstract). Journal of Hepatology 2003;38(Suppl 2):168.	**yes**	**1**	No	

117	**Sarin 1999**	Sarin SK, Guptan RC, Thakur V. Efficacy of interferon alone or ribavirin interferon combination in HCV related cirrhosis and chronic hepatitis patients: a randomized controlled trial (abstract). Hepatology 1999;30(4 Pt 2):634A.	**yes**	**1**	No	
118	**Scotto 1996**	Scotto G, Fazio V, Tantimonaco G. Pilot study of a short course of ribavirin and alpha interferon in the treatment of chronic active hepatitis C not responding to alphainterferon alone. Italian Journal of Gastroenterology 1996;28 (9):505–11. [MEDLINE: 97278061; : PMID: 9131395]	**yes**		Yes	**33**
119	**Scotto 2003**	Scotto G, Campanozzi F, D'Adduzio A, Grimaldi M, Fazio V. Interferon-alpha (IFN alpha) daily dose versus IFN alpha plus ribavirin for treatment-naive chronic hepatitis c patients infected by genotype 1b. BioDrugs 2003;17(4):281–6.	**yes**		Yes	**34**
120	**Senturk 2003**	Senturk H, Ersoz G, Ozaras R, Kaymakoglu S, Bozkaya H, Akdogan M, et al. Interferon-alpha2b induction treatment with or without ribavirin in chronic hepatitis C: a multicenter, randomized, controlled trial. Digestive Diseases and Sciences 2003;6:1124–9.	**yes**		Yes	**35**
121	**Senturk 2003**	Senturk H, Ersoz G, Ozares R, Kaymakoglu S, Bozkaya H, Akdogan M, et al. Interferon-alfa induction treatment with or without ribavirin in chronic hepatitis C: A multicenter randomized controlled study (abstract). Hepatology 2002;36(4):579A.	**no**		No	
123	**Shiffman 2000**	Shiffman ML, Hofmann CM, Gabbay J, Luketic VA, Sterling RK, Sanyal AJ, et al. Treatment of chronic hepatitis C in patients who failed interferon monotherapy: effects of higher doses of interferon and ribavirin combination therapy. American Journal of Gastroenterology 2000;95(10):2928–35.	**yes**		Yes	**36**
124	**Shobokshi 2003**	Shobokshi O, Serebour EK, Skakni L, Tantawi A, Mohamed A, Sandokji A, et al. PEG-INF alfa-2b as a monotherapy or in combination with ribavirin significantly improve end of treatment response rate in HCV genotype 4 chronic active hepatitis (CAH) patients (abstract). Hepatology 2002;36(4(Pt 2 of 2)):362A.	**no**		No	
125	**Shobokshi 2003**	Shobokshi O, Serebour F, Skakni L, Tantawi A, Dinish T, Al Quaiz M, et al. Efficacy of pegylated (40 KDA) IFN alfa-2a (Pegasys) plus ribavirin in the treatment of hepatitis C genotype 4 chronic active in Saudi Arabia (abstract). Journal of Hepatology 2002;36 Suppl 1:129A.	**no**		No	
126	**Shobokshi 2003**	Shobokshi OA, Serebour FE, Skakni L, Al-Jaser N, Tantawe AO, Sabah A, et al. Combination therapy of Peginterferon alfa-2a and ribavirin significantly enhances sustained virological and biochemical response rate in chronic hepatitis C genotype 4 in Saudi Arabia (abstract). Hepatology 2003;38(4 Suppl 1):636A.	**no**		No	
127	**Shobokshi 2003**	Shobokshi OA, Serebour FE, Skakni L, Al-Jaser N, Tantawe AO, Sabah A, et al. Pegylated interferon alfa-2a (40KDA) as a monotherapy or in combination with ribavirin significantly improves end of treatment response rate in hepatitis C virus genotype 4 chronic active hepatitis patients (abstract). Saudi Medical Journal 2003;24(Suppl2):S92.	**yes**	**1**	No	
128	**Sievert 2003**	Sievert W, Batey R, Mollison L, Pianko S, McDonald J, Marinos G, et al. Induction interferon and ribavirin for retreatment of chronic hepatitis C patients unresponsive to interferon alone. Alimentary Pharmacology & Therapeutics 2003;17(9):1197–204.	**yes**	**6**	No	
129	**Sostegni 1998**	Sostegni R, Ghisetti V, Pittaluga F, Marchiaro G, Rocca G, Borghesio E, et al. Sequential versus concomitant administration of ribavirin and interferon alfa-n3 in patients with chronic hepatitis C not responding to interferon alone: results of a randomized, controlled trial. Hepatology 1998;28(2):341–6. [MEDLINE: 98359166; : PMID: 9695995]	**yes**		Yes	**37**
130	**Sostegni 1998**	Sostegni R, RizzettoM,Ghisetti V, Pittaluga F, Marchiaro G, Rocca G, et al. Sequential versus concomitant administration of ribavirin and interferon alfa-n3 in patients with chronic hepatitis C not responding to interferon alone. Results of a randomized controlled trial. Journal of Hepatology 1998;28 Suppl 1:122.	**no**		No	
131	**Taliani 1999**	Taliani G, Badolato MC, Pasquazzi C, Boddi V, Gaeta GB, Stornaiuolo G, et al. Early HCV-RNA clearance during daily administration of interferon in combination with ribavirin in naive HCV patients (abstract). Journal of Hepatology 2000;32 Suppl 2:179.	**yes**	**1**	No	
132	**Taliani 1999**	Taliani G, Badolato MC, Pasquazzi C, Gaeta GB, Stornaiuolo G, Corti G, et al. Daily IFN and ribavirin administration induces early HCV-RNA clearance (abstract). Hepatology 1999; Vol. 30, issue 4 Pt 2:632A.	**no**		No	

**133**	**Tempini 2001**	Tempini S, Grimoldi D, Mondazzi L, Cattaneo C, Bellobuono A, Ideo GM, et al. IFN alpha-con 1 + riba combination therapy in naive difficult-to-treat patients affected by chronic hepatitis C (abstract). Journal of Hepatology 2001;34 Suppl 1:147–8.	**yes**	**1**	No	
**134**	**Toccaceli 1997**	Toccaceli F, Grimaldi M, Rosati S, Palazzini E, Laghi V. Ribavirin plus human leucocyte interferon alpha for the treatment of interferon resistant chronic hepatitis C: a controlled trial. Hepatology Research 1997;8(2):106–12.	**yes**		Yes	**38**
**135**	**Trippi 2000**	Trippi S, Di Gaetano G, Soresi M, Cartabellotta F, Vassallo R, Carroccio A, et al. Interferon-alfa alone versus interferonalfa plus ribavirin in patients with chronic hepatitis C not responding to previous interferon-alfa treatment. BioDrugs 2000;13(4):299–304.	**yes**		Yes	**39**
**136**	**Vandelli 2000a**	Vandelli C, Renzo F, Vecchi C, Ventura E, Tisminetzky S. Retreatment of chronic hepatitis C patients with previous non-response to interferon alone (abstract). Journal of Hepatology 2000;32 Suppl 2:106.	**yes**	**1**	No	
**137**	**Verbaan 2002**	Verbaan HP, Widell HEA, Bodeson TL, Lindgren SC. High sustained response rate in patients with histologically mild (low grade and stage) chronic hepatitis C infection. A randomised, double blind, placebo-controlled trial of interferon alpha-2b with or without ribavirin. European Journal of Gastroenterology and Hepatology 2002;14:627–33.	**yes**		Yes	**40**
**138**	**Wood 1998**	Wood MM, Malet PF, Jones A, Prebis M, Harford W, Lee WM. High dose interferon alfa-2b (IFN) with or without ribavirin (RIB) for chronic hepatitis C in non-responders to standard therapy (abstract). Hepatology 1998 Suppl 4;28: 283A.	**yes**	**1**	No	
