# Supplementary material for: A statistical method for removing unbalanced trials with multiple covariates in meta-analysis
Source: PLoS One. 2023 Dec 15;18(12):e0295332. doi: 10.1371/journal.pone.0295332 (PMC10723740; doi:10.1371/journal.pone.0295332)
Supplement: Attachment — Submitted filename: comments.pdf [file pone.0295332.s012.pdf]

Review Comments to the Author on "A statistical method to assess and to adjust for covariate imbalance in meta-analysis"

July 30, 2023

This paper presents a novel method for identifying unbalanced trials in meta-analysis, with the aim of achieving combinability. This concept holds significant importance and has been frequently discussed in meta-analysis literature. The proposed approach relies on a backward reduction procedure utilizing the combined Anderson-Darling test, enabling efficient detection and elimination of unbalanced trials. Moreover, the method can be seen as an extension of the one proposed by Aiello, Attanasio, and Tinè (2011).

Major Comments:

- The original paper and the previous work by Aiello et al. used the notation of A_{kN}. It will be better if you either use this fashion to write your formula here (e.g, adding one more subscript here to represent h^{th} PLV) or write your A^2 explicitly if you mean the different thing.

- The k-sample Anderson-Darling criterion assumes independent samples. However, it is possible that we can encounter dependent studies (See Lin and Sullivan (2009) and Han et al. (2016)). Can your method address this scenario? Any comments or discussions will be appreciated.

- Any statistical properties about A_c^2 or T_c? For example, what kind of distributions will they follow?

- On page 16, it is evident that the results obtained are significantly different. However, since the true effect sizes of the datasets are unknown, it is challenging to draw definitive conclusions. To establish the efficacy of the proposed process and demonstrate its potential in yielding more reliable results, I suggest conducting several small simulation studies. These simulations can assess various aspects, such as the accuracy of effect size estimation, reduction in variance, and other relevant metrics.

- The backward reduction procedure is a very interesting method for detecting biased studies within a meta-analysis, where T_c statistics will be used as a criterion. However, when there is a limited number of studies available, the reliability of T_c statistics may decrease due to reduced statistical power. Do you have any comments or discussions about this point?

- The primary focus of this paper lies in studies with higher incidence rates, which typically involve more common events. However, it is essential to consider the potential impact of the proposed method on rare events as well, where background incidence rates can be lower, even below 0.5%. See Bhaumik et al. (2012) and Zhang et al. (2023).

Minor Comments:

- I can't find a definition of A_h^2.

- I have trouble finding Table 2-4, please check the label indexes.

- Any comments on Fig 4?

- I like the tables (Table I & II) in Aiello, Attanasio, and Tinè (2011) to explain how ECDF was calculated. Can you include some similar tables here? You may include them in the Appendix.

References

Aiello, Fabio, Massimo Attanasio, and Fabio Tinè (Sept. 2011). "Assessing covariate imbalance in meta-analysis studies". en. In: *Statistics in Medicine* 30.22, pp. 2671–2682.

Bhaumik, Dulal K. et al. (June 2012). "Meta-Analysis of Rare Binary Adverse Event Data". en. In: *Journal of the American Statistical Association* 107.498, pp. 555–567.

Han, Buhm et al. (May 2016). "A general framework for meta-analyzing dependent studies with overlapping subjects in association mapping". en. In: *Human Molecular Genetics* 25.9, pp. 1857–1866.

Lin, Dan-Yu and Patrick F. Sullivan (Dec. 2009). "Meta-Analysis of Genomewide Association Studies with Overlapping Subjects". In: *The American Journal of Human Genetics* 85.6, pp. 862–872.

Zhang, Ming et al. (May 2023). "Bayesian estimation and testing in random-effects meta-analysis of rare binary events allowing for flexible group variability". In: *Statistics in Medicine* 42.11, pp. 1699–1721.
